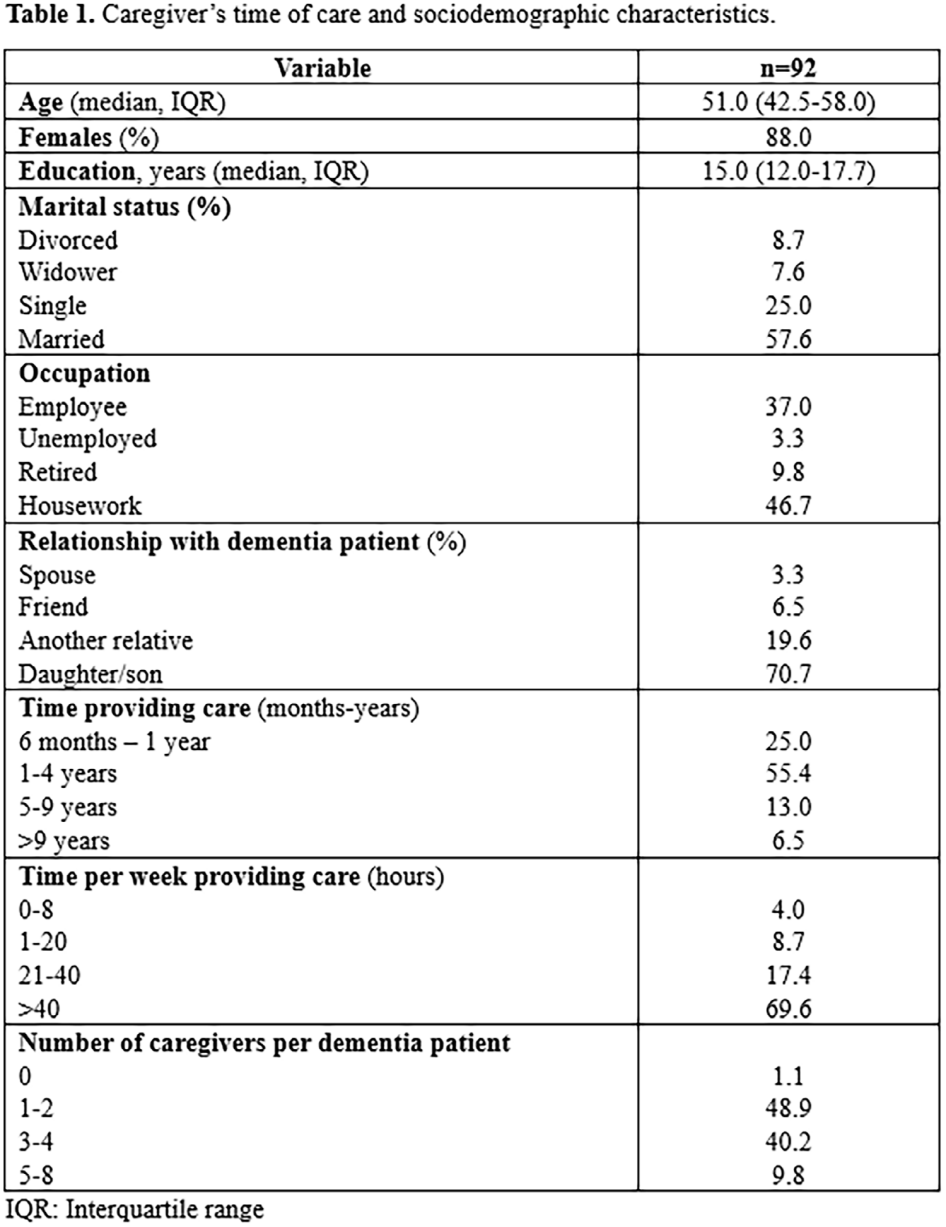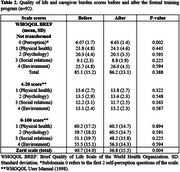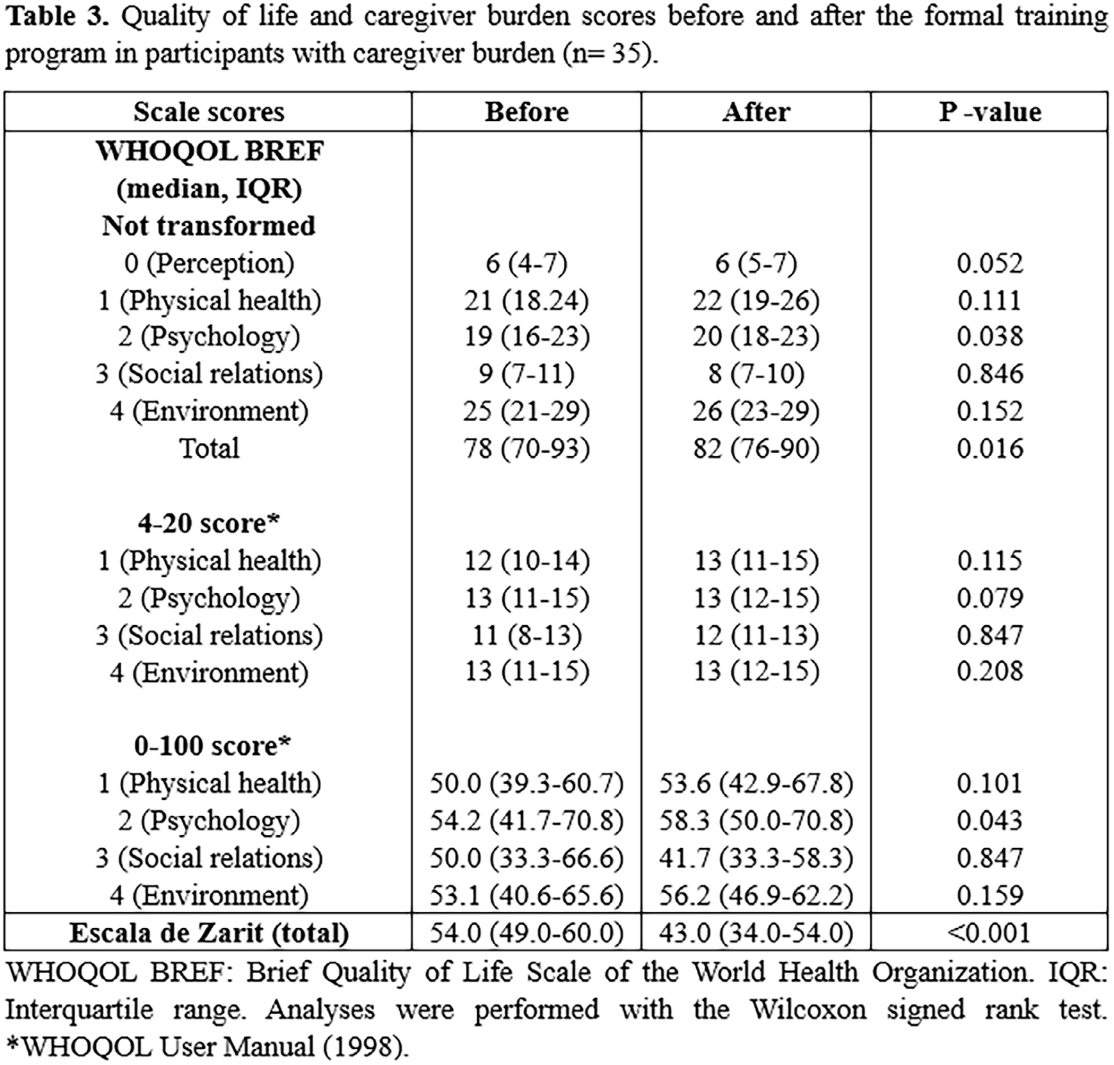# Effects of formal training on dementia patient's caregivers quality of life and care burden in an outpatient clinical setting

**DOI:** 10.1002/alz70858_103345

**Published:** 2025-12-25

**Authors:** Sara Gabriela Yeverino‐Castro, Estefania Elizabeth Abundis‐Márquez, Ana Cecilia Muñoz‐Díaz, Roxana Debanhi Limón‐Castillo, Célica Raquel Gonzalez‐Galván, José Luis Valdez‐Cruz, Andrés Garza‐Dávila, Sergio Guillermo Mendez‐Salas, Ricardo Salinas‐Martínez, Rocio Morales‐Delgado

**Affiliations:** ^1^ CHRISTUS – Center of Excellence and Innovation, San Pedro Garza García, NL, Mexico; ^2^ Dr. José E. González University Hospital, Monterrey, NL, Mexico

## Abstract

**Background:**

Dementia affects 50 million people worldwide, causing disability, dependence, and death. At least 90 percent of dementia patients exhibit behavioral and psychological symptoms which affect their quality of life (QoL) and that of their caregivers.

**Method:**

To evaluate the impact of a training program on QoL and caregiver burden (CB), a quasi‐experimental study involving primary caregivers for dementia patients who attended an outpatient geriatric clinic from January 2024 to January 2025, was performed. A 1‐month caregiver formal training was conducted online using written and videotaped materials. Participants' sociodemographic and clinical characteristics, CB and QoL, assessed with the Zarit and WHOQoL‐BREF scales, respectively, were described at baseline and after the intervention. Individuals with a Zarit score >47 points were classified as with CB. Parametric and non‐parametric statistics were used to analyze data. A sub‐analysis was performed on individuals with CB.

**Result:**

Caregiver's time of care and sociodemographic characteristics are described in Table 1. From a total of 92 individuals, median age was 51.0 (IQR: 42.5‐58.0) years and 88.0% were women. Most caregivers were dementia patients' daughters or sons (70.7%), married (57.6%), dedicated to housework (46.7%) or were still employed (37.0%), and committed >40 hours of care to their relative (69.6%) in a 1–4‐year timespan (55.4%). The most prevalent cognitive diagnosis amongst dementia patients was Alzheimer's disease (47.8%) and the most used pharmacological treatment was antidementia‐drugs (64.1%) followed by antidepressants (46.7%). Caregiver's QoL and CB scale scores before and after the intervention are presented in Table 2. Both QoL (WHOQoL‐BREF perception subdomain) and CB amongst all participants were significantly improved after the training program (*p* = 0.002 and *p* = 0.004, respectively). Sub‐analysis results are presented in Table 3. Similarly, when assessing individuals with a CB diagnosis a significant improvement in caregiver's QoL (psychology subdomain and total score) and CB (*p* = 0.038, *p* = 0.016, and *p* <0.001, respectively) was also observed.

**Conclusion:**

This study provides preliminary evidence on psychoeducational interventions that can possibly improve caregiver's well‐being, promote resilience, and equip them with better knowledge about dementia patients. Eventually, these types of interventions benefit both caregivers and older adults with dementia, leading to better overall outcomes.